# A New Inactivated Coxsackievirus B2 Vaccine: Biological Properties, Immunogenicity, and Protective Effects in Mice

**DOI:** 10.3390/vaccines14040290

**Published:** 2026-03-24

**Authors:** Zhaoyang Chu, Changzeng Feng, Ming Zhang, Xiang Li, Hengli Yang, Jiansheng Liu, Shaohui Ma

**Affiliations:** Institute of Medical Biology, Chinese Academy of Medical Sciences & Peking Union Medical College, Kunming 650118, China; ttu25662@gmail.com (Z.C.); fengchangzeng@163.com (C.F.); xinxiangyujing@foxmail.com (M.Z.); llllxx0317@163.com (X.L.); hengli01@126.com (H.Y.); ljsh3300@163.com (J.L.)

**Keywords:** coxsackievirus B2, biological characteristic, animal model, inactivated vaccine, immunogenicity

## Abstract

**Background**: Coxsackievirus B2 (CVB2) causes a range of diseases, including hand, foot, and mouth disease; myocarditis; acute flaccid paralysis; meningitis; and encephalitis. However, no specific antiviral drugs or vaccines are currently available for CVB2. **Methods**: We used plaque purification, virus titre determination, and serial passaging to screen and identify an inactivated CVB2 vaccine candidate strain, KM31-C05, which exhibited high viral titres and good genetic stability. Comprehensive biological characterization of this candidate strain was performed, including phylogenetic analysis, virulence assessment in BALB/c mice, one-step growth curve analysis, optimization of the multiplicity of infection, as well as determination of viral load, pathological evaluation, and immunohistochemical analysis in tissues of BALB/c suckling mice post-challenge. An experimental inactivated vaccine was prepared using KM31-C05 to evaluate its immunogenicity and protective efficacy. **Results**: The viral titres of KM31-C05 reached 10^8^ CCID50/mL. After 20 serial passages, only three amino acid mutations were identified (VP3-G165V, VP1-N84K, and VP1-D129N). Although the two VP1 mutations were located in surface-exposed loops, the strain maintained high neutralizing titres across passages, indicating good genetic stability. However, whether these sites affect virulence and replication requires further investigation. Phylogenetic analysis revealed that this strain belonged to genotype C, which is consistent with the strains circulating in mainland China in recent years. The experimental inactivated vaccine prepared from KM31-C05 induced effective neutralizing antibodies (1:128–1:256) in BALB/c mice and provided complete protection to suckling mice against lethal challenge with this CVB2 strain in maternal antibody protection experiments. **Conclusions**: KM31-C05 demonstrates potential as a CVB2 vaccine candidate in China and provides a theoretical basis for the development of a CVB2 vaccine.

## 1. Introduction

Enteroviruses (EVs) belong to the family *Picornaviridae* and genus *Enterovirus*. To date, the *Enterovirus* genus has been classified into 15 species: *Enterovirus A* through *L* (EV-A to EV-L) and *Rhinovirus A* through *C* (RV-A to RV-C). Among these, EV-A, EV-B, EV-C, EV-D, and all three *Rhinovirus* species are known to infect humans, causing a diverse range of clinical manifestations [1]. Coxsackievirus B2 (CVB2), the focus of this study, belongs to the *Enterovirus B* species, which was first isolated as the prototype strain Ohio-1 in 1947 [2]. CVB2 is a non-enveloped, single-stranded, positive-sense RNA virus with a diameter of ~30 nm. Its genome is ~7400 nucleotides long and is flanked by a 5′- and a 3′-untranslated region. The coding region contains a single open reading frame that encodes a polyprotein that is subsequently cleaved into functional proteins. This polyprotein is divided into the P1, P2, and P3 regions. The P1 region encodes four structural proteins (VP1–VP4). While VP1 contains the major antigenic determinants, significant antigenic sites have also been characterized in the VP2 and VP3 proteins of various enteroviruses. The P2 and P3 regions encode seven non-structural proteins (2A–2C and 3A–3D, respectively) [3,4].

CVB2 causes a broad spectrum of human diseases, ranging from mild to life-threatening. It is associated with hand, foot, and mouth disease (HFMD), characterized by fever, sore throat, and vesicular rash on the hands, feet, and mouth, primarily affecting young children. CVB2 can also cause severe conditions, including myocarditis (potentially leading to heart failure); acute flaccid paralysis; aseptic meningitis (with symptoms such as headache, neck stiffness, and photophobia); and encephalitis (which may result in altered mental status, seizures, and neurological sequelae) [5,6,7,8,9,10,11]. The circulation of CVB2 is typically endemic, with outbreaks documented in several countries. CVB2 accounts for 1.5–6.0% of annually reported EV infections [12]. Surveillance data indicate that the virus is prevalent across diverse geographic regions and can cause severe clinical manifestations. For example, in 2019, a 42-year-old male patient with CVB2 infection developed multi-organ involvement, necessitating distal pancreatectomy, splenectomy, renal dialysis, veno-arterial extracorporeal membrane oxygenation, and mechanical ventilation [13]. In 2022, an outbreak of CVB2-associated meningitis was reported among children in Israel [14,15].

At present, no specific vaccines or antiviral agents have been approved for the prevention or treatment of CVB2 infections, and clinical management primarily relies on supportive care. While significant efforts in enterovirus vaccine development have led to the commercial availability of vaccines against EV71 and polioviruses [16,17], these monovalent vaccines do not confer cross-protection against other serotypes, including CVB2 [18]. Consequently, there remains an urgent need for the development of a vaccine specifically targeting CVB2 to fill this prophylactic gap. There are safety concerns associated with live-attenuated vaccines, such as the potential for attenuated pathogens to mutate and partially regain virulence, which can result in severe disease among immunocompromised individuals; therefore, inactivated vaccines remain the preferred approach for the development of a CVB2 vaccine [19,20].

The CVB2 prototype strain, Ohio-1, was initially isolated in the USA in 1947 [2,21]. Currently, a universally standardized genotyping system for CVB2 has yet to be established. In this study, we adopted the classification criteria described in the previous literature [22], which defines distinct genotypes based on a nucleotide sequence divergence of at least 15% in the VP1 region. According to this criterion, CVB2 has evolved into six genotypes (A–F) characterized by distinct geographic distributions: genotype A (USA, Taiwan); genotype B (Australia, France, UK, USA); genotype C (primarily China); genotype D (India, Madagascar, South Africa, Russia); genotype E (China, Thailand, Australia, USA, South Korea); and genotype F (Japan, USA). Studies have shown that cross-neutralization among different genotypes of EVA71 can vary [23], and vaccine strains may provide limited protection against heterologous genotypes. Currently, the only multivalent CVB vaccine candidate, including the CVB2 serotype, that has entered clinical trials is being developed in Finland. However, the genotype of the vaccine strain used differs from that of strains prevalent in China, potentially limiting its efficacy against circulating CVB2 strains in China. In this study, we identified a CVB2 vaccine candidate strain, KM31-C05, which is capable of growing to high titres in Vero cells and exhibits good genetic stability. This strain belongs to a predominant genotype in China, and its biological properties were thoroughly characterized. The experimental vaccine prepared from the KM31-C05 strain elicited high titres of homologous neutralizing antibodies against other homologous or the same strains, suggesting that KM31-C05 could serve as a promising CVB2 vaccine candidate.

## 2. Materials and Methods

### 2.1. Cells and Viruses

The CVB2 strains KM135, KM294, KM501, KM509, and KM31 (corresponding sequences have been deposited in the GenBank database under accession numbers PV035077, PV035078, PV853927, PV853928, and MW365443, respectively) were isolated from faecal samples of hand, foot, and mouth disease patients in the Yunnan Region of China between 2010 and 2022. These strains are preserved at the Institute of Medical Biology, Chinese Academy of Medical Sciences (Table S1). Vero cells (Vero 76, ATCC CRL-1587) were used for CVB2 propagation and vaccine production. The cells were cultured in Dulbecco’s modified Eagle’s medium supplemented with 10% newborn calf serum (MINHAI BIO, Beijing, China), 2% L-glutamine, 100 IU/mL penicillin, and 100 µg/mL streptomycin. All cultures were maintained at 37 °C in a 5% CO_2_ atmosphere.

### 2.2. Plaque Purification

To obtain monoclonal CVB2 strains, each isolate was subjected to three rounds of plaque purification. The viral stock was serially diluted 10-fold, and dilutions at 10^−3^–10^−5^ were inoculated onto confluent Vero cell monolayers. The cells were incubated at 37 °C in a 5% CO_2_ atmosphere for 1 h to allow for viral adsorption. After incubation, the supernatant was removed, and the cells were gently washed twice with phosphate-buffered saline (PBS). Subsequently, a 1:1 (*v*/*v*) mixture of 2× Minimum Essential Medium (Gibco, Grand Island, NY, USA) and 1.5% methylcellulose in cell culture-grade water was added, and the cultures were incubated at 35 °C in a 5% CO_2_ incubator. Plaque formation was monitored daily using light microscopy. Individual plaques were selected and inoculated into fresh Vero cell cultures, which were observed for several days for the development of a cytopathic effect (CPE). Once CPE was observed, the supernatant was collected, and viral RNA was extracted. The virus was identified as CVB2 by reverse transcription polymerase chain reaction (RT-PCR), sequencing, and sequence alignment. The same procedures were repeated for the second and third rounds of plaque purification.

### 2.3. RT-PCR and Sequencing

Viral RNA was extracted from the supernatant of infected cells using the Axygen Body Fluid Viral DNA/RNA Miniprep Kit (Axygen, Hangzhou, China). Amplification of the VP1 gene and entire genome was performed using the PrimeScript One Step RT-PCR Kit version 2 (Takara, Dalian, China). Sequencing was carried out on an ABI 3130 Genetic Analyzer (Applied Biosystems, Foster City, CA, USA). The primer sequences used for amplification and sequencing are listed in Table 1. EV genotyping was performed using the Enterovirus Genotyping Tool. Available online: https://www.rivm.nl/mpf/typingtool/enterovirus/ (accessed on 7 April 2023) [24].

### 2.4. Phylogenetic Analysis and Sequence Alignment

Nucleotide sequences of the complete VP1 region (846 nt) were obtained for all study strains and aligned with reference CVB2 sequences from GenBank using Geneious 9.0.2 software with default parameters (MAFFT algorithm, accuracy-oriented settings). Phylogenetic analysis was conducted using MEGA version 7.0. A neighbor-joining tree was constructed based on the Kimura 2-parameter model with 1000 bootstrap replicates. The results yielded bootstrap support values > 80%.

### 2.5. Infectious Titres

Viral infectious titres were determined using the microtissue culture technique [25]. Following serial 10-fold dilutions, virus samples were inoculated onto Vero cells in 96-well plates (eight replicates per dilution) and incubated at 37 °C in a 5% CO_2_ atmosphere for 7 days. The 50% cell culture infectious dose (CCID_50_) was calculated using the Reed–Muench method.

### 2.6. Genetic Stability

The four purified strains, KM135-C01, KM294-C02, KM31-C05, and KM509-C01, were serially passaged 20 times to assess their genetic stability. Viral RNA was extracted from passages P1, P5, P10, P15, and P20, followed by whole-genome amplification and sequencing (Table 2). The infectious titres were determined using the Reed–Muench method.

### 2.7. Growth Kinetics of KM31-C05 Strain

The KM31-C05 strain, which exhibited high viral titres and genetic stability, was selected as the vaccine candidate strain for one-step growth curve analysis. To ensure a synchronized infection where virtually all cells are infected simultaneously, a high multiplicity of infection (MOI) of 10 was employed. KM31-C05 was inoculated onto confluent Vero cell monolayers (90–95% confluence) at an MOI of 10. The virus was adsorbed at 37 °C for 1 h, after which the supernatant was removed, and the cells were washed three times with PBS. Subsequently, 1 mL Minimum Essential Medium was added to each well, and the cultures were incubated at 35 °C in a 5% CO_2_ atmosphere. Cells were harvested at 0, 1, 2, 4, 6, 12, 20, 28, 36, and 44 h post-infection (hpi). Replicate wells were set up at each time point to ensure the reliability of the results. To further optimize viral production parameters, Vero cells were infected at varying seeding MOIs (1, 0.1, 0.01, and 0.001). The time required to reach 95% CPE and the corresponding peak viral titres were recorded. All samples underwent three freeze–thaw cycles before titration using the Reed–Muench method.

### 2.8. Animal Challenge

According to previously published protocols [22,26,27], 3-day-old BALB/c mice were selected for challenge experiments. Viral inoculation was performed via intraperitoneal injection, with four challenge doses (10^5.5^ CCID_50_, 10^4.5^ CCID_50_, 10^3.5^ CCID_50_, and 10^2.5^ CCID_50_) to identify the optimal challenge dose. Mice in the control group received 30 µL uninfected cell lysate per animal and were housed separately from the virus-inoculated groups. All mice were monitored for 14 days, and clinical symptoms were scored as follows: 0, healthy; 1, lethargy/inactivity; 2, weight loss; 3, limb weakness, sparse fur, hunchback posture, or impaired mobility; 4, moribund or death [28].

### 2.9. Histopathology and Immunohistochemistry

Three-day-old BALB/c mice were inoculated intraperitoneally with 10^4.5^ CCID_50_ of KM31-C05. When the clinical score reached 4, animals were anesthetized and killed for tissue collection. Tissues were fixed in 4% formalin, processed routinely, and embedded in paraffin. Sections were stained with haematoxylin and eosin for histopathological evaluation. For immunohistochemistry, deparaffinized sections underwent antigen retrieval, followed by incubation with a rabbit polyclonal antibody against CVB2 (Institute of Medical Biology, Chinese Academy of Medical Sciences) at a dilution of 1:1500, and treatment with a goat anti-rabbit secondary antibody. Positive signals were visualized with a DAB chromogenic kit (Servicebio, Wuhan, China).

### 2.10. Viral Load of CVB2 in Different Organs

At 6, 12, 24, 48, 72, and 96 hpi, mice were sacrificed, and tissues were collected without systemic perfusion. The collected tissues—including blood, pancreas, spleen, kidney, liver, small intestine, heart, lung, forelimb muscle, hindlimb muscle, and brain—were weighed and homogenized using a high-throughput tissue homogenizer (TISSUELYSER II; Qiagen, Duesseldorf, Germany). Total RNA was extracted from the homogenates using TRIzol reagent (Invitrogen, Carlsbad, CA, USA). Viral RNA was quantified by RT-qPCR using the One Step PrimeScript™ RT-PCR Kit for Perfect Real-Time PCR (Takara). The specific primers and probe used for RT-qPCR were as follows: B2-qpF(5′-GGCAATACCAGCACTCACTG-3′), B2-qpR(5′-GTAGTTGTGCACGTGTCTGG-3′), and B2-Probe (FAM-ACGGGTCACACCTCGCAAGTCA-BHQ1). For absolute quantification, a specific RNA standard was prepared by in vitro transcription of a CVB2 standard plasmid. A standard curve was generated using ten-fold serial dilutions of the RNA standard to enable the quantitative determination of viral RNA copies per mg of tissue.

### 2.11. Preparation of Inactivated CVB2 Vaccine

To evaluate the immunogenicity of the vaccine candidate, an immunization study was conducted using eight-week-old BALB/c mice. Mice were immunized via the intraperitoneal (i.p.) route with two different doses of the inactivated vaccine (1 μg or 3 μg in 100 μL per mouse). The CVB2 experimental inactivated vaccine was prepared as described previously [29,30]. CVB2 strain KM31-C05 at passage 5 (P5) was inoculated onto Vero cells at an MOI of 0.01. When the CPE exceeded 90%, the viral supernatant was harvested. After three freeze–thaw cycles, the virus suspension was filtered through a 0.45-μm membrane filter to remove cellular debris. Formaldehyde solution (Sigma–Aldrich, St. Louis, MO, USA) was added to a final concentration of 1:4000 (*v*/*v*), and the mixture was incubated at 37 °C for 48 h to achieve virus inactivation. The inactivated virus was blind-passaged three times in Vero cells, and the absence of CPE was considered indicative of successful inactivation. The inactivated virus suspension was concentrated using a 100 kDa PES membrane (Millipore, Billerica, MA, USA) and further purified using a Capto Core 400 gel filtration column. The protein concentration of the purified inactivated virus was determined using the Pierce BCA Protein Assay Kit (Rockford, IL, USA). Finally, aluminium hydroxide adjuvant (2024-01-S, the Institute of Medical Biology, Chinese Academy of Medical Sciences, China) was added to formulate the inactivated vaccine, with the final aluminium concentration adjusted to 0.35 mg/mL, which is lower than the FDA limit of 0.85 mg aluminium per vaccine dose.

### 2.12. Immunogenicity of the Inactivated CVB2 Vaccine

Eight-week-old female BALB/c mice were randomly divided into three groups of five. Two immunizations were administered via intraperitoneal injection on days 0 and 21. Two experimental groups received CVB2 inactivated vaccine doses of 1 and 3 µg, respectively, while the control group was injected intraperitoneally with an equal volume of a mixture of aluminium adjuvant and PBS. Blood samples were collected on days 0, 14, 35, and 49, and sera were isolated. Neutralizing antibody titres were determined as described previously [31,32]. Briefly, twofold serial dilutions of heat-inactivated serum samples (starting at a 1:4 dilution, which was defined as the lower limit of detection) were mixed with an equal volume of virus suspension containing 100 CCID_50_ and incubated at 37 °C for 1 h. The mixtures were then added to Vero cell monolayers in 96-well plates (two replicates per serum dilution) and incubated at 37 °C in a 5% CO_2_ atmosphere for 7 days. Neutralizing antibody titres were calculated as the highest serum dilution that completely inhibited the cytopathic effect.

### 2.13. Maternal Antibody Protection

Eight-week-old female BALB/c mice were intraperitoneally injected with 100 µL of CVB2 inactivated vaccine containing 3 µg antigen and 35 µg aluminium adjuvant, while control mice received an equal volume of a mixture of aluminium adjuvant and PBS via the same route. Mice were immunized with a two-dose schedule, consisting of a prime immunization on Day 0 and a boost on Day 21. Two weeks after initial immunization, male and female mice were co-housed for mating. Neonatal mice aged 3 days were intraperitoneally challenged with the homologous CVB2 KM31-C05 strain at a dose of 10^4.5^ CCID_50_ following birth. The body weight and survival status of the neonatal mice were recorded daily for 14 consecutive days post-challenge.

### 2.14. Statistical Analysis

All data analyses were performed using GraphPad Prism version 9.0.0 (San Diego, CA, USA). Statistical methods included one-way analysis of variance (ANOVA) and *t*-tests. A *p*-value < 0.05 was considered statistically significant.

## 3. Results

### 3.1. Primary Characteristics of Viral Isolates

**Figure 1:**
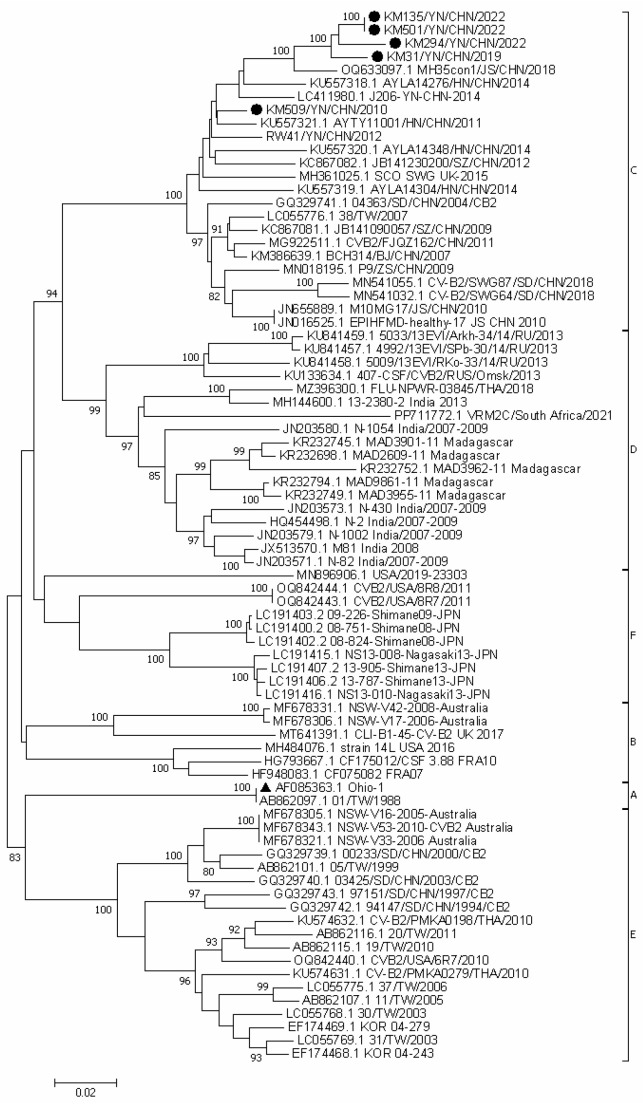
Phylogenetic analysis was conducted on the complete VP1 gene sequence (846 nt) of CVB2, incorporating five isolates from this study along with 74 representative CVB2 strains retrieved from GenBank. The phylogenetic testing used 1000 bootstrap replications and the Kimura 2-parameter model. The results yielded bootstrap support values > 80%. ▲ CVB2 prototype strain, ● CVB2 strains isolated in this study.

All CVB2 strains in this study were isolated from Vero cells and exhibited the characteristic cytopathic effect (CPE) typical of enteroviruses. According to the criterion that nucleotide differences > 15% in the EV VP1 gene define different genotypes within the same serotype, CVB2 can be classified into six genotypes (A–F). All five CVB2 strains in this study belonged to genotype C, which is consistent with the genotypes currently circulating in mainland China (Figure 1). The nucleotide and amino acid sequence similarities of the VP1 gene among these five strains ranged from 92.6% to 100% and 97.9% to 100%, respectively. Compared to the prototype strain Ohio-1, these five strains shared 82.6–83.1% nucleotide similarities and 97.5–98.2% amino acid similarities. KM501 strain failed to produce plaques after the second round of plaque purification, preventing the isolation of a monoclonal clone. The other four strains that underwent plaque purification were designated as KM135-C01, KM294-C02, KM31-C05, and KM509-C01. The viral titres of these strains were 10^8.04^, 10^6.54^, 10^7.96^, and 10^7.25^ CCID_50_/mL, respectively (Figure S1). Except for KM294-C02, all other strains had titres > 10^7^ CCID50/mL. Among them, KM135-C01 exhibited the highest titre, followed by KM31-C05.

### 3.2. Screening of Challenge Doses

To determine the appropriate challenge dose, 3-day-old BALB/c mice were intraperitoneally injected with different dilutions of CVB2/KM31 ranging from 10^5.5^ to 10^2.5^ CCID_50_ per mouse in 10-fold serial dilutions. The mice showed dose-dependent clinical manifestations. In the 10^5.5^ CCID_50_ group, deaths occurred as early as 1 day post-infection (dpi), with mortality reaching 100% by 3 dpi. The 10^4.5^ CCID_50_ group began showing symptoms at 2 dpi, with deaths starting at 4 dpi and reaching 100% mortality by 5 dpi; deaths were clustered in time. In the 10^3.5^ and 10^2.5^ CCID_50_ groups, symptoms appeared between 3 and 4 dpi, and mortality was more dispersed over time, with both groups reaching 100% mortality by 10 dpi (Figure S2). Considering both clinical symptoms and time to death, a challenge dose of 10^4.5^ CCID_50_ per mouse was selected for subsequent experiments. Based on these experimental conditions, three independent replicate experiments were conducted at different times to validate the stability of the model. Mice exhibited significant clinical signs, including weight loss and anorexia, between 2 and 3 dpi. Deaths began from 3 to 5 dpi, with mortality reaching 100% by 6 to 7 dpi (Table S2), demonstrating good stability and reproducibility of the infection model.

### 3.3. Virulence of Four CVB2 Strains

**Figure 2:**
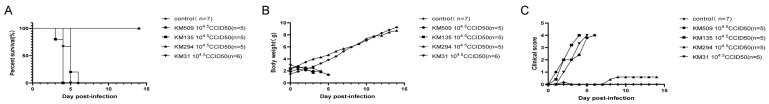
Virulence of different CVB2 isolates in BALB/c mice. Three-day-old BALB/c mice were divided into four groups and received intraperitoneal injections of four CVB5 isolates (KM135-C01, KM294-C02, KM31-C05, and KM509-C01) at a dose of 10^4.5^ CCID_50_ per 30 μL per mouse. Control groups were inoculated with the same volume of virus-free cell supernatant. (**A**) Percent survival; (**B**) Body weight; (**C**) Clinical score.

To assess the virulence of the four plaque-purified CVB2 strains (KM135-C01, KM294-C02, KM31-C05, and KM509-C01), 3-day-old BALB/c mice were intraperitoneally inoculated with the same dose (10^4.5^ CCID_50_ per mouse) of each viral strain. Clinical symptoms were monitored daily for 14 consecutive days (Figure 2). Mice challenged with the KM294-C02 strain exhibited minimal clinical signs, with only one mouse displaying sparse fur throughout the observation period, and no deaths were observed. In contrast, mice in the KM135-C01, KM31-C05, and KM509-C01 groups began dying between 3 and 6 dpi, with mortality reaching 100% in each group. Therefore, the KM294-C02 strain demonstrated the lowest virulence, while no significant differences in virulence were observed among the other three strains.

### 3.4. Genetic Stability of KM31-C05 Strain

The VP1 gene sequences of four CVB2 strains at different passages (P1, P5, P10, P15, and P20) were sequenced. Except for the KM31-C05 strain, the other strains exhibited double peaks in chromatograms after P5 or P10, which was presumed to be caused by mutations. Therefore, full-genome sequencing and alignment were performed for KM31-C05 at P1, P5, P10, P15, and P20. Three amino acid mutations were identified over 20 passages: one mutation occurred in the VP3 gene (VP3-165 G→V, from P5 to P20), and two mutations were located in the VP1 gene (VP1-84 N→K and VP1-129 D→N, both observed at P15 and P20) (Table 2). Despite these mutations, the nucleotide and amino acid sequence homologies of the whole genome among different passages of KM31-C05 remained high, ranging from 99.9% to 100% and 99.7% to 100%, respectively. Additionally, viral infectious titres were measured for P1, P5, P10, P15, and P20, yielding 10^7.96^, 10^8.25^, 10^7.79^, 10^7.92^, and 10^8.29^ CCID_50_/mL, respectively (one-way ANOVA, *p* > 0.05; Figure S3), indicating that the KM31-C05 strain maintained high and stable titres throughout passage. Considering its high-yield characteristics in cell culture, potent pathogenicity in the neonatal mouse model, and excellent genetic stability, the KM31-C05 strain was selected as the vaccine candidate for subsequent studies.

### 3.5. Growth Characteristics of KM31-C05 Strain

**Figure 3:**
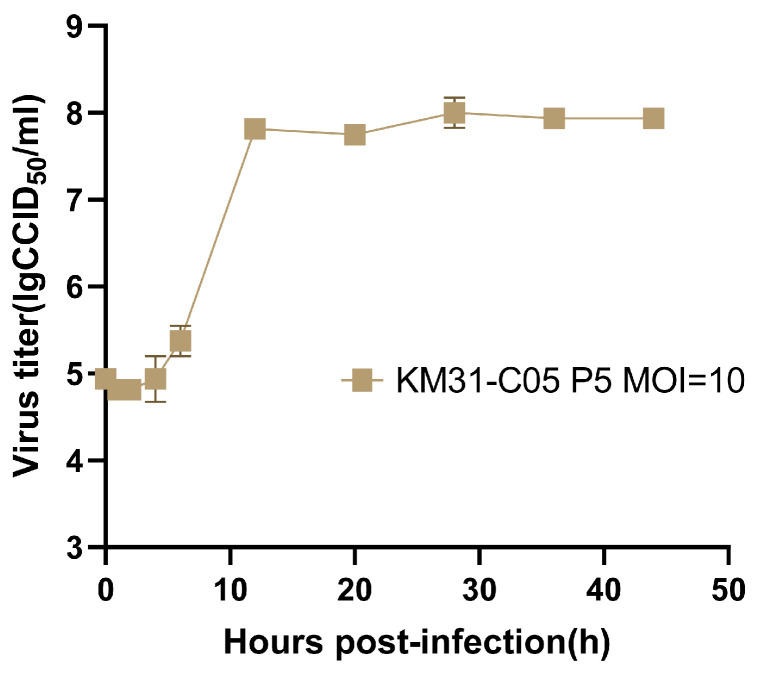
One-step growth curve of KM31-C05 in Vero cells. The virus titres of KM31-C05 were measured by Reed–Muench at 0, 1, 2, 4, 6, 12, 20, 28, 36, and 44 hpi with an MOI of 10. Data are shown as mean ± SEM.

To investigate the replication characteristics of the working seed strain KM31-C05 (P5), a one-step growth curve was constructed at a high MOI of 10. The viral titre remained stable during the eclipse phase (0–4 hpi), followed by a logarithmic increase from 4 to 12 hpi. The viral yield reached a plateau after 12 hpi, indicating a rapid and efficient replication cycle in Vero cells (Figure 3). Subsequently, we evaluated the impact of different seeding MOIs on harvest timing and final viral yields to determine the optimal parameters for vaccine production. As shown in Table 3, the time required to reach 95% CPE was inversely proportional to the seeding MOI, ranging from 24 hpi (MOI = 1) to 60 hpi (MOI = 0.001). While all tested MOIs yielded high viral titres (>10^7.8^ CCID50/mL), an MOI of 0.01 provided the most favorable balance between high-titre virus amplification (10^8.25^ CCID50/mL) and practical production efficiency. Therefore, an MOI of 0.01 and a harvest window associated with 95% CPE were selected for subsequent inactivated vaccine manufacturing.

### 3.6. Histopathological and Immunohistochemical Analyses of KM31-C05-Infected Mice

**Figure 4:**
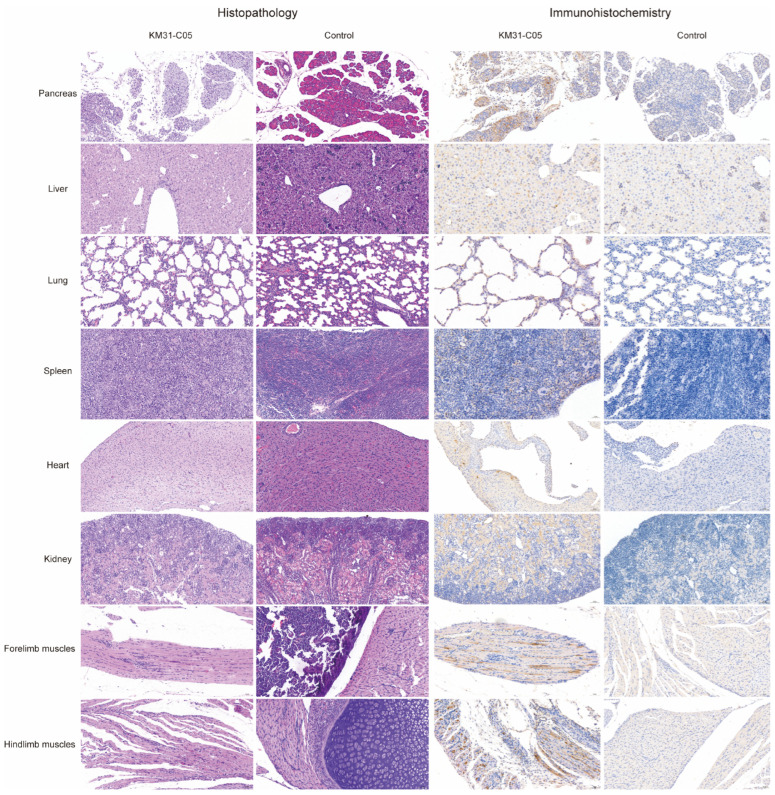
Histopathology and immunohistochemistry of tissues from KM31-C05 P5-infected BALB/c mice. Three-day-old BALB/c mice were inoculated intraperitoneally with KM31-C05 P5. When infected mice reached a clinical score of 4, they were killed prior to haematoxylin and eosin staining and immunohistochemistry. The experiments were performed in triplicate, and one representative result is shown.

The liver showed lymphocytic infiltration around the central vein, and extensive hepatocellular steatosis with round vacuoles of varying sizes in the cytoplasm. Spleen tissue had widespread extramedullary haematopoiesis in the red pulp, abundant neutrophilic infiltration, and multinucleated giant cell infiltration. Pancreatic tissue showed focal acinar necrosis with nuclear fragmentation, connective tissue proliferation, and substantial inflammatory cell infiltration; the interstitium exhibits mild oedema and a sparse arrangement of connective tissue with some lymphocytic infiltration. In the lungs, upper limb muscles, and lower limb muscles, there was a small amount of inflammatory cell infiltration. Cardiac and kidney tissues showed cytopathic changes. No significant pathology was observed in the brain or small intestine (Figure 4). Immunohistochemical analysis showed that CVB2 antigen was detected in the exocrine pancreas, spleen, liver, forelimb muscle, hindlimb muscle, kidney, heart, and lung (Figure 4), whereas no viral antigen was found in the brain and small intestine of infected mice. In the control group, no histopathological changes or viral antigen expression were observed in any tissues.

### 3.7. Viral Loads in Different Organs of KM31-C05-Infected Mice

**Figure 5:**
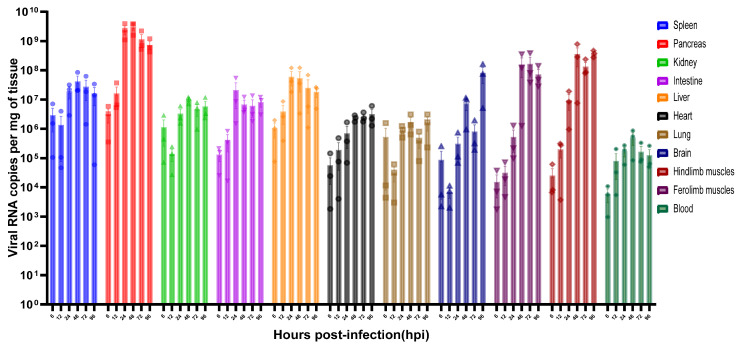
Tissue viral loads in KM31-C05 P5-infected mice were measured at multiple times. Three-day-old mice received intraperitoneal inoculation of 30 μL KM31-C05 P5 (10^4.5^ CCID_50_ per mouse). Pancreas, spleen, kidney, liver, small intestine, heart, lung, forelimb muscle, hindlimb muscle, brain, and blood were collected at 6, 12, 24, 48, 72, and 96 hpi (*n* = 3, per time point). Viral loads were quantified by RT-qPCR and are presented as mean ± SEM (*n* = 3).

Viral RNA in tissue samples was quantified by RT-qPCR using the One Step PrimeScript™ RT-PCR Kit (Takara), with results expressed as viral RNA copies per mg of tissue. At 6 hpi, viral loads in several organs were approximately 10^4^ viral RNA copies per mg of tissue. Given the time required for systemic dissemination and tissue entry following intraperitoneal injection, these early RNA levels primarily reflect the distribution of the input virus rather than active viral replication. Significant increases in viral RNA were observed in subsequent time points. The pancreas consistently exhibited the highest burden, peaking at 10^9.49^ viral RNA copies per mg of tissue at 48 hpi. The liver reached a peak of 10^7.78^ viral RNA copies per mg of tissue, while the brain and limb muscles reached peak loads between 10^7.97^ and 10^8.57^ viral RNA copies per mg of tissue at 72 or 96 hpi (Figure 5).

### 3.8. Immunogenicity of Inactivated CVB2 Vaccine

**Figure 6:**
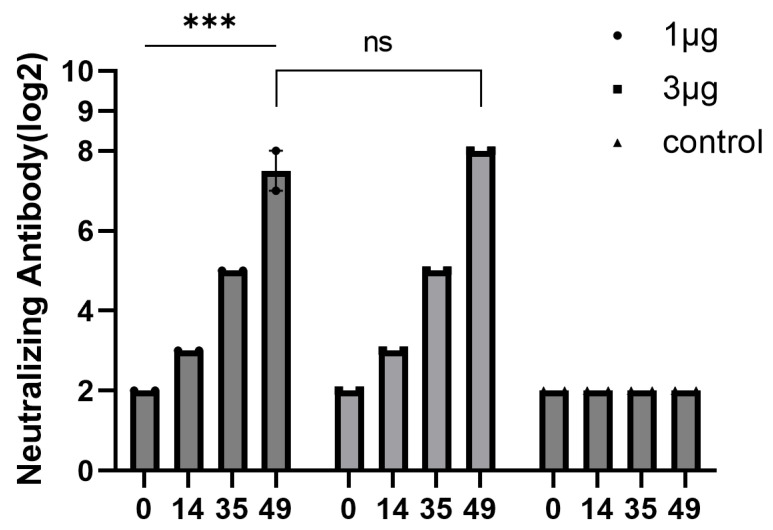
Dynamic characteristics of neutralizing antibody responses induced by different doses of inactivated CVB2 vaccine in BALB/c mice. Two immunizations were administered via intraperitoneal injection on days 0 and 21. Two experimental groups received CVB2 inactivated vaccine doses of 1 and 3 µg, respectively, while the control group was injected intraperitoneally with an equal volume of a mixture of aluminium adjuvant and PBS. The x-axis labels 0, 14, 35, and 49 represent four post-immunization blood collections at 0, 14, 35, and 49 days after the first immunization, respectively. Sera from five mice in each group were pooled to form one biological sample, and *n* = 2 such pooled samples were analyzed per group. Data are presented as individual data points and mean ± SEM. Statistical significance was analyzed using t-tests and one-way ANOVA (*** *p* < 0.001; ns, non-significant).

Female BALB/c mice were immunized twice with the CVB2 inactivated vaccine prepared from the KM31-C05 P5 strain. On day 28 after the second immunization, neutralizing antibody titres in the 1 and 3 µg dose groups were significantly increased, reaching 1:192 and 1:256, respectively (Figure 6). No clinical abnormalities, such as ruffled fur or reduced activity, were observed in any vaccinated mice during the experimental period, suggesting that the vaccine was well-tolerated in the BALB/c mouse model. The difference in neutralizing antibody titres between the two dose groups was not significant (*p* > 0.05). Antisera induced by the CVB2 inactivated vaccine demonstrated significant neutralizing activity against multiple clinical isolates. The cross-neutralization titres of antisera induced by the 1 µg dose of vaccine against other strains (KM135-C01, KM509-C01, and KM294-C02) ranged from 1:96 to 1:256. However, no neutralizing activity was observed against the CVB2 prototype strain Ohio-1, with neutralization titres < 1:4 (Table S3).

### 3.9. Maternal Antibody Protection

**Figure 7:**
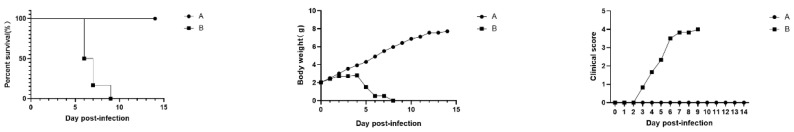
Protective effect of maternal CVB2 antibodies. To evaluate the passive protective efficacy of maternal antibodies, eight-week-old female BALB/c mice were immunized twice (Day 0 prime, Day 21 boost) with the CVB2 inactivated vaccine. The control group received an equal volume of aluminium adjuvant and PBS following the same schedule. After delivery, three-day-old neonatal mice from both groups were challenged with the homologous CVB2 KM31-C05 strain. A: Three-day-old neonatal BALB/c mice (*n* = 6) born to dams immunized twice with the inactivated CVB2 vaccine (Day 0 prime, Day 21 boost) were challenged with the homologous CVB2 KM31-C05 strain. B: Three-day-old neonatal BALB/c mice (*n* = 6) born to dams that received aluminium adjuvant plus PBS on the same schedule were challenged with the homologous CVB2 KM31-C05 strain.

To evaluate the protective effect of maternal antibodies, 8-week-old female BALB/c mice were immunized twice with the CVB2 inactivated vaccine, while the control group received intraperitoneal injections of an aluminium adjuvant and PBS mixture following the same schedule. After delivery, 3-day-old neonatal mice were challenged with the homologous CVB2 KM31-C05 strain. All neonatal mice in the control group died within 9 days post-challenge, whereas those born to vaccinated dams exhibited no clinical symptoms and demonstrated continuous weight gain (Figure 7). These findings indicate that the CVB2 inactivated vaccine induced maternal antibodies that conferred complete protection to neonatal mice against challenge with the homologous CVB2 strain.

## 4. Discussion

To date, only two vaccine development programs have involved CVB2, both of which focused on polyvalent inactivated vaccines covering all six CVB serotypes. The first study, conducted in the 1990s, demonstrated that the polyvalent CVB vaccine provided protection against all six serotypes; however, mild pancreatitis was still observed in some animals during challenge experiments [33]. Achievement of optimal protection required three doses of the vaccine. Although this regimen elicited neutralizing antibodies, the antibody levels varied among subjects [33,34]. This study did not proceed to subsequent clinical trials. More recently, a formalin-inactivated polyvalent CVB vaccine was developed and evaluated in BALB/c mice, NOD mice, and SOCS-1 transgenic mouse models. This vaccine showed broad protection across multiple serotypes, effectively preventing CVB-induced myocarditis, pancreatitis, and diabetes without mortality. Its immunogenicity was also confirmed in non-human primates [35,36,37]. In a phase I clinical trial (NCT04690426), this vaccine demonstrated good tolerability and robust immunogenicity [38], supporting further clinical development. However, the CVB2 strain used in this vaccine was isolated in Finland, and phylogenetic analysis based on the VP1 gene revealed considerable genetic distance between epidemic strains from different regions. Notably, the predominant European CVB2 strains and those circulating in China belong to different genotypes. Following the isolation of the prototype CVB2 strain Ohio-1 in 1947, six distinct genotypes (A–F) have been identified and characterized based on their genetic diversity and geographic distribution [22]. The Chinese epidemic strains predominantly belong to genotypes C and E. Genotype E includes strains isolated between 1994 and 2011 from China, Thailand, Australia, the USA, and South Korea, while genotype C primarily comprises strains isolated in China from 2004 to 2022. Additionally, genotype A includes two strains isolated in the USA (1947) and Taiwan, China (1988); genotype B consists of strains from Australia, France, UK, and the USA isolated between 2006 and 2017; genotype F encompasses strains isolated in Japan and the USA from 2008 to 2013; and genotype D includes isolates from India, Madagascar, South Africa, and Russia collected between 2007 and 2021. The CVB2 strains used in this study all belonged to genotype C, consistent with recent epidemic strains in China. VP1 is the major antigenic determinant of EVs. The VP1 amino acid sequence differences between the five CVB2 strains in this study and recent Chinese mainland strains deposited in GenBank ranged from 0.7% to 2.8%. In contrast, the differences compared to strains from other countries were more pronounced, ranging from 0.7% to 8.9%.

Differences exist in cross-neutralization among various genotypes of EVs. In cross-neutralization assays of different EV71 genotypes, vaccines prepared from the C4 genotype strain elicited significant cross-neutralizing responses against other C4 genotype strains, while neutralizing antibody titres against genotype A strains were the lowest [23]. Our findings align with this pattern; antisera induced by our inactivated CVB2 vaccine (based on a currently circulating Genotype C strain) failed to neutralize the historical prototype Ohio-1 strain (Genotype A). This indicated that CVB2 may exhibit significant antigenic drift over time or space. Consequently, the polyvalent CVB vaccine developed in Finland might offer limited protection against the distinct genotypes currently prevailing in China due to these divergent amino acid sequences and antigenic profiles. Therefore, it is necessary to develop a vaccine specifically targeting the current Chinese strains to effectively prevent CVB2 infections.

In the process of vaccine development, the selection of an appropriate cell line is critical. Currently, the two commercially available EV vaccines—EV71 and poliovirus vaccines –are both produced using Vero cells or KMB17 (human diploid embryonic lung fibroblast cell line). Vero cells are among the most widely used cell lines for viral vaccine production. Besides EVs, they are also used in the manufacture of vaccines against rabies and Japanese encephalitis viruses, and are a suitable and safe platform for the production of human inactivated vaccines [39]. The CVB2 strains were adaptively cultured and plaque-purified in Vero cells, followed by titre determination. KM135-C01 and KM31-C05 exhibited high infectious titres of 10^8.04^ CCID_50_/mL and 10^7.96^ CCID_50_/mL, respectively; KM509-C01 showed a moderate titre of 10^7.25^ CCID_50_/mL, while KM294-C02 had the lowest titre at 10^6.54^ CCID_50_/mL. Considering vaccine production costs, strains with higher titres are preferentially selected as vaccine candidates. Subsequent genetic stability analysis revealed that, except for KM31-C05, other strains exhibited double peaks in sequencing chromatograms during passage, which is presumed to result from mutations arising during viral propagation. The presence of these double peaks compromised accurate sequence determination for these strains. Virulence testing of these strains showed that KM294-C02 had the lowest virulence, whereas KM135-C01, KM31-C05, and KM509-C01 showed no significant differences in virulence. Taking into account viral infectious titre, virulence, and genetic stability, KM31-C05 was selected as the vaccine candidate for further study. RNA viruses possess high genetic variability due to the lack of proofreading capability in their RNA-dependent RNA polymerases, resulting in elevated replication error rates [40]. Mutations occurring during vaccine production may affect immunogenicity and efficacy; thus, evaluation of vaccine strain genetic stability is a crucial component of vaccine quality control. Over 20 serial passages, KM31-C05 accumulated three amino acid substitutions (VP3-165 G→V for P5 to P20, VP1-84 N→K for P15 and P20, and VP1-129 D→N for P15 and P20). However, antibodies against KM31-C05 P5 neutralized the different generations of viruses (P1, P5, P10, P15, and P20) with neutralizing antibody titres ≥ 1:64. Previous studies reported two amino acid mutations in the CVB5 vaccine candidate strain KM299-L05 during passage, and three amino acid mutations in the CVB1 vaccine candidate strain KM7-X29 [31,32] did not affect their immunogenicity. KM31-C05 maintained a consistently high infectious titre of ~10^8^ CCID_50_/mL across P1, P5, P10, P15, and P20. This indicated that KM31-C05 had good genetic stability, which makes it a promising candidate for a CVB2 vaccine.

Subsequently, more biological characterizations of the KM31-C05 vaccine candidate strain were conducted. Animal models are essential tools in vaccine development. In this study, an animal model was established by intraperitoneally challenging 3-day-old BALB/c mice with a dose of 10^4.5^ CCID_50_ per mouse of the KM31-C05 strain, which displayed a stronger tropism for the liver and pancreas. Pathological lesions were also detected in the spleen, kidney, heart, lung, forelimb muscle, and hindlimb muscle. CVB2 can also induce damage in several organs in humans [11], such as pancreatitis, myocarditis, and meningitis. However, pathological studies on CVB2 remain limited. In the study by Hong et al. [41], two Korean isolates (CB2/04/279, derived from stool of a patient with acute myocarditis and heart failure, and CB2/04/243, isolated from a throat swab of a patient with aseptic meningitis) along with the prototype CVB2 strain Ohio-1 were analysed for the pathological changes they induced in 4-week-old male BALB/c mice. Mice infected with CB2/04/279 and Ohio-1 developed myocarditis and extensive pancreatitis, whereas such lesions were not observed in the CB2/04/243 group. The challenge in this experiment was administered via intraperitoneal injection, and no neuropathological analysis was performed. In a separate study by Ushioda et al. [2], newborn ddY mice were intracerebrally inoculated with the prototype CVB2 strain Ohio-1 to investigate neuropathological features following infection. They observed dome-shaped heads, cerebral cortical loss, and ventricular enlargement within 3 weeks, with pathological changes restricted to the cortical region. The pathogenic features exhibited by CVB2 infection are influenced by factors such as the viral clinical isolate, route of inoculation, and animal strain.

A one-step growth curve of the KM31-C05 strain was generated, and the optimal MOI was determined. According to the growth curve, the strain exhibited a latent period from 0 to 4 h, a rapid logarithmic growth phase between 4 and 12 h, followed by a plateau phase after 12 h. When infected at MOI 0.01, CPE appeared within a moderate time frame, and high viral titres were achieved in culture. This MOI is suitable for scale-up virus production, consistent with findings from related studies on CVB1 and CVB5 [31,32].

To assess the immunogenicity of the CVB2 inactivated vaccine prepared from the KM31-C05 P5 strain, neutralization assays were performed using antisera induced by the vaccine. The neutralizing antibody titres reached 1:192 and 1:256 in the 1 and 3 µg dose groups, respectively. Cross-neutralization assays against other CVB2 strains (KM135-C01, KM509-C01, and KM294-C02) showed neutralizing antibody titres ranging from 1:96 to 1:256. Previous studies on poliovirus vaccines have indicated that neutralizing antibody titres ≥ 1:8 provided clinical protection [42], while the protective threshold for EV71 inactivated vaccines was ≥1:32 [43]. The experimental CVB2 vaccine in the present study showed promising immunogenicity. In the maternal antibody protection experiment, neonatal mice born to vaccinated dams were fully protected against lethal CVB2 challenge, exhibiting no clinical symptoms and zero mortality. It is noteworthy that maternal antibody transfer in mice occurs primarily via the yolk sac splanchnopleure and lactation, which differs from the predominantly placental transfer in humans. Therefore, the complete protection observed in 3-day-old neonates—despite their relatively lower initial antibody levels compared to later stages—further underscores the high efficacy and rapid protective capacity of the maternal antibodies induced by the KM31-C05 vaccine.

This study also has limitations: First, the use of neonatal mice, whose immune systems are underdeveloped, poses challenges in husbandry and observation. Second, the maternal antibody challenge experiment lacked exposure to multiple viral strains. Third, the characterization of inactivated vaccine antigens was based solely on chromatographic purification profiles and electron microscopy observations (Figures S4 and S5). Finally, the safety assessment of this candidate vaccine did not include a detailed evaluation of potential toxicities or histopathological changes following immunization. In summary, this study successfully screened a predominant circulating CVB2 strain in China as a vaccine candidate. This strain possesses favourable infectious titre, virulence, and genetic stability. The experimental inactivated vaccine derived from this strain demonstrates good immunogenicity and protective efficacy, providing a strong foundation for future CVB2 vaccine development and the creation of polyvalent CVB vaccine platforms.

## Figures and Tables

**Table 1:** Amplification and sequencing primers of whole genome sequence.

Primer	Sequence (5′→3′)	Site
B-OAS	GGTGCTCACTAGGAGGTCYCTRTTRTARTCYTCCCA	3601–3564
B-OS	GGYTAYATNCANTGYTGGTAYCARAC	2303–2328
E201F	TTAAAACAGCCTGTGGGTTG	1–20
B21r	ACAGACTGCCCACTGGTG	515–498
B22f	GCCATCCAGTCAGCAATAGAGCA	634–656
B23f	CGGCCAAGATAACGCCAAAGAGT	1390–1412
B21R	CACTGCCAATAGTGTCAGC	2516–2498
B22F	CGCGACACTCGCTTTATCACAC	2417–2438
B24f	CAAGGCTAGTAACGTGAAC	3235–3253
B25r	ACAGCTGCTCTTGGTCAC	4277–4260
B22R	TCGGTCACGAGCATGTCCAATG	4974–4953
B23F	TCGTCCTTGCCTCCACTAATGC	4698–4719
B26f	CAGCATTTGAATTCGCAGTG	5373–5392
B27r	TGGTGGGAATTGCACAAGTAG	6738–6718
B28f	TGACGGTCATCTCATAGCC	6592–6610
E208R	ACCGAATGCGGAGAATTTAC	7404–7385

Note: F/f is the upstream primer, R/r is the downstream primer, B-OS is the upstream primer, B-OAS is the downstream primer.

**Table 2:** Amino acid variations in the whole genome of different generations of the KM31-C05 strain.

Virus	Amino Acid Site		
	VP3-165	VP1-84	VP1-129
KM31-C05-P1	G	N	D
KM31-C05-P5	V	N	D
KM31-C05-P10	V	N	D
KM31-C05-P15	V	K	N
KM31-C05-P20	V	K	N
CVB2 Ohio-1	V	N	D

Note: Amino acid positions were referenced to the whole genome sequence of the KM31-C05-P1 strain. The full names corresponding to each amino acid abbreviation are: G (Glycine), V (Valine), N (Asparagine), K (Lysine) and D (Aspartate).

**Table 3:** Optimization of CVB2 KM31-C05 harvest parameters at varying MOIs.

Seeding MOI	Time to 95% CPE (hpi)	Viral Titre (Log_10_ CCID50/mL)
1	24	7.81
0.1	36	8.06
0.01	52	8.25
0.001	60	8.30

## Data Availability

All data supporting the findings of this study are included in the article and its Supplementary Materials. If additional information is required, please contact the corresponding author directly.

## Supplementary Materials

The following supporting information can be downloaded at https://www.mdpi.com/article/10.3390/vaccines14040290/s1. Figure S1: Virus titre of the different CVB2 isolates; Figure S2: Screening of challenge doses; Figure S3: Titres of KM31-C05 across passage generations; Figure S4. Chromatographic purification profile of the inactivated CVB2 vaccine antigen. Figure S5. Morphological characterization of the purified CVB2 vaccine antigen via electron microscopy. Table S1: Information on five CVB2 isolates from Yunnan Province in the study; Table S2: The repeatability and stability of the CVB2-challenged neonatal mouse model. Table S3. Cross-neutralization titres of antisera induced by 1 µg of inactivated CVB2 vaccine against different strains.

## Abbreviations

The following abbreviations are used in this manuscript:

CVB2	Coxsackievirus B2
EVs	Enteroviruses
PBS	Phosphate-buffered saline
CPE	Cytopathic effect
RT-PCR	Reverse transcription polymerase chain reaction
CCID50	50% cell culture infectious dose
MOI	Multiplicity of infection
hpi	Hours post-infection
dpi	Days post-infection
