# Supplementary material for: A New Inactivated Coxsackievirus B2 Vaccine: Biological Properties, Immunogenicity, and Protective Effects in Mice
Source: Vaccines (Basel). 2026 Mar 24;14(4):290. doi: 10.3390/vaccines14040290 (PMC13119634; doi:10.3390/vaccines14040290)
Supplement: Supplementary file 1 [file vaccines-14-00290-s001.zip › vaccines-4137500-supplementary.pdf]

Supplementary Information

Table S1. Information on five CVB2 isolates from Yunnan Province in the study.

Strain	Year of separation	Sample Type	Diagnosis	Cell line
KM135	2022	faeces	Mild HFMD	Vero
KM294	2022	faeces	Mild HFMD	Vero
KM501	2022	faeces	Mild HFMD	Vero
KM31	2019	faeces	Mild HFMD	Vero
KM509	2010	faeces	Severe HFMD	Vero

Table S2. The repeatability and stability of the CVB2-challenged neonatal mouse model.

Experiment	Number of mice	Onset of symptoms (d)	Onset of death (d)	The time of all mice died (d)	Mortality ratio (%)
1	6	2	3	6	100
2	5	3	5	7	100
3	8	2	3	7	100

Table S3. Cross-neutralization titers of antisera induced by 1 μg of inactivated CVB2 vaccine against different strains.

strains	KM135-C01	KM294-C02	KM509-C01	Ohio-1
cross-neutralization titre	1 ： 128	1 ： 96	1 ： 256	<1 :4

Note: Antisera were collected from 8-week-old BALB/c mice immunized with two doses of 1 μg of the inactivated CVB2 vaccine (Day 0 prime and Day 21 boost). Neutralization titers were determined by a neutralization assay.

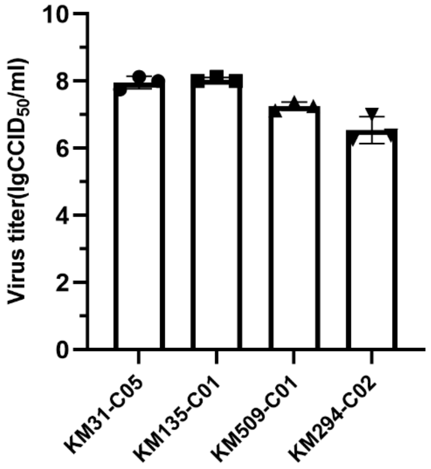

Figure S1. Virus titer of the different CVB2 isolates. Viral titers of the CVB2 strains (KM135-C01, KM294-C02, KM31-C05, and KM509-C01) were determined after three rounds of plaque purification. Titers are expressed as $\log_{10}$ CCID50/mL.

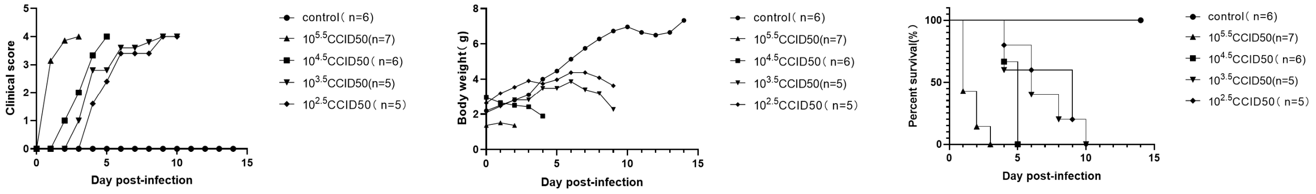

Figure S2. Screening of Challenge Doses. Three-day-old BALB/c mice were intraperitoneally (i.p.) injected with 10-fold serial dilutions of CVB2/KM31, ranging from $10^{5.5}$ to $10^{2.5}$ CCID50 per mouse, to determine the optimal challenge dose.

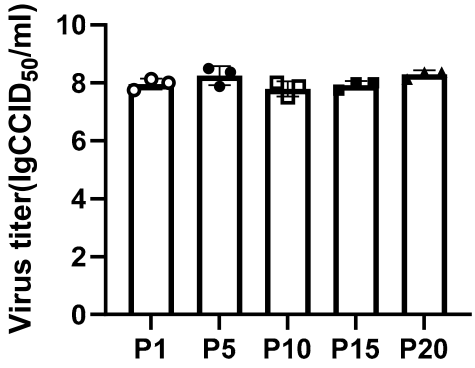

Figure S3. Titers of KM31-C05 across passage generations. Infectious titers were measured for passage levels P1, P5, P10, P15, and P20. No significant variation in viral titers was observed across 20 serial passages (one-way ANOVA, $p > 0.05$).

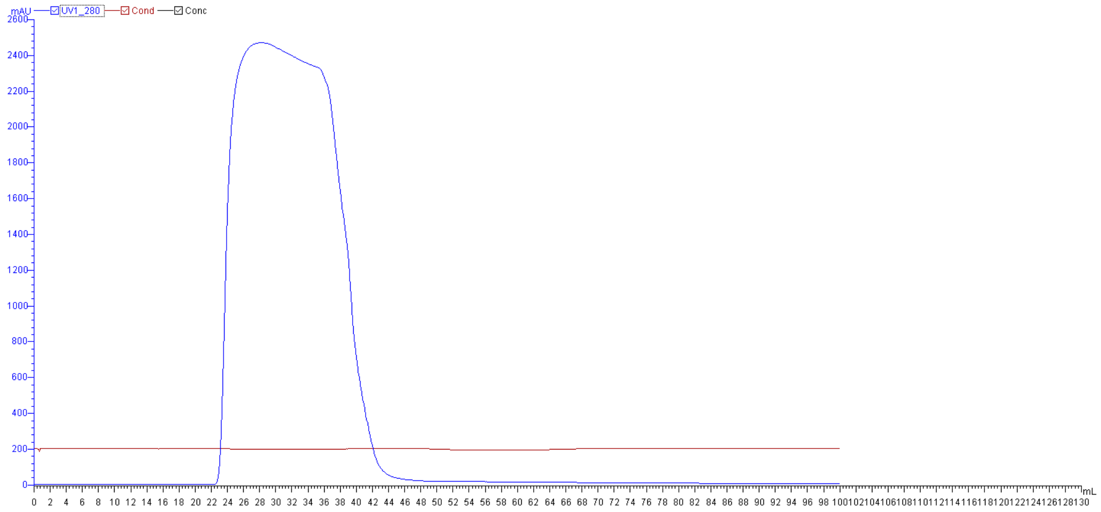

Figure S4. Chromatographic purification profile of the inactivated CVB2 vaccine antigen. The concentrated CVB2 virus suspension was purified using a Capto Core 400 gel filtration column.

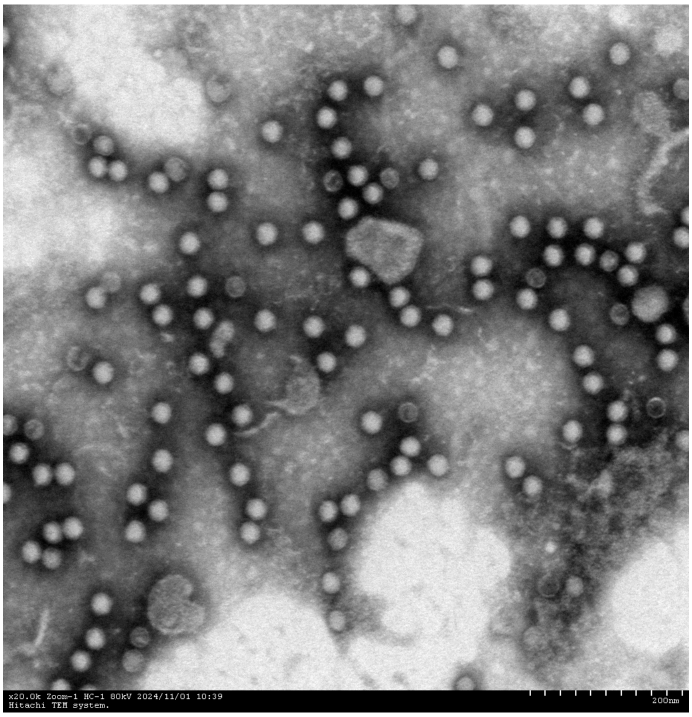

Figure S5. Morphological characterization of the purified CVB2 vaccine antigen via electron microscopy. The particles exhibit typical enterovirus morphology with a spherical structure and a diameter of approximately 30 nm.
